# Disseminating clinical study results to trial participants in Ethiopia: insights and lessons learned

**DOI:** 10.1186/s12936-020-03279-5

**Published:** 2020-06-08

**Authors:** Tamiru S. Degaga, Sophie Weston, Tedla T. Tego, Dagimawie T. Abate, Ashenafi Aseffa, Adugna Wayessa, Ric N. Price, Asrat Hailu, Kamala Thriemer

**Affiliations:** 1 College of Medicine & Health Sciences, Arba Minch University, Arba Minch, Ethiopia; 2 Global and Tropical Health Division, Menzies School of Health Research and Charles Darwin University, Darwin, NT Australia; 3 Arba Minch General Hospital, Arba Minch, Ethiopia; 4 Ethiopian Public Health Institute, Addis Ababa, Ethiopia; 5 Mahidol-Oxford Tropical Medicine Research Unit, Faculty of Tropical Medicine, Mahidol University, Bangkok, Thailand; 6 Centre for Tropical Medicine and Global Health, Nuffield Department of Medicine, University of Oxford, Oxford, UK; 7 College of Health Sciences, Addis Ababa University, Addis Ababa, Ethiopia

**Keywords:** Clinical trial, Result dissemination, Participant experience, Participant feedback, Patient feedback, Malaria

## Abstract

International regulatory authorities and funders require that research be disseminated promptly and appropriately to all involved stakeholders. However, following completion of clinical trials participants often either do not receive any feedback or materials provided are not appropriate for the context. The investigators of a multicentre anti-malarial clinical trial (the IMPROV study) conducted a dissemination meeting at one of the study sites in Ethiopia; trial participants and medical staff were provided feedback on the study results. This report summarizes the dissemination strategies adopted by the investigators, including a plain language visual aid and simple communication techniques. Lessons learned are reported with a discussion on the operational challenges to dissemination of clinical trials in resource limited settings.

## Background

Dissemination of study findings is central to completion of clinical trials and is mostly aimed at facilitating translation of evidence into policy and practice. Scientists report their results to a range of audiences, including other academics and healthcare professionals, policy makers, funders and the general public. There are strong incentives for researchers to comply with these communication expectations either to further their careers through presentation at academic conferences and publications [1] or because of the nature of their agreements with the funders of their research [2–5]. Incentives to disseminate study results to trial participants are less compelling. In a large survey among authors of trials indexed in PubMed, only 5% to 20% of investigators had disseminated or planned to disseminate study results to their trial participants [6]. The main barriers to dissemination were researchers’ perception about the limited interest of participants, difficulties in reaching participants following trial completion, a lack of early planning and respective support for the activities, a lack of incentives and different cultural expectations, concerns over potential misunderstanding and misrepresentations of the nature of inconclusive or negative results, and a general unfamiliarity with the methods that could be used to ensure dissemination was constructive and would achieve the desired results [6].

The Declaration of Helsinki provides ethical responsibilities pertaining to clinical trials [7], and these are reiterated by clinical trial regulations in the European Union [8], the US [9] and Canada [10], all of which require summary results to be provided to study participants in plain language. Dissemination of results to participants is an ethical imperative [7], but has additional benefits, such as improving public trust in medical research and encouraging participation in future studies, thus contributing to overall health literacy with potentially improved uptake of research findings into policy and practice [11].

Many trial investigators engage patient groups and communities in the design of trials to improve acceptability and enhance recruitment [12–14]. Community Advisory Boards are often established to increase community engagement and oversee study conduct [15, 16]. However, little is known about the best format and mode of delivery to provide feedback to participants after the completion of a trial. This is particularly challenging for clinical trials in low- and middle-income countries where patient education levels are lower, and participants are often less empowered.

This article presents the lessons learned from a dissemination meeting with participants who had been enrolled into a clinical trial for radical cure of vivax malaria in Ethiopia between 2016 and 2017 [17, 18].

### The IMPROV study

The IMPROV study was designed to determine the efficacy and safety of a short-course high dose primaquine regimen for the radical cure of vivax malaria. It enrolled 2388 patients at eight sites located in Indonesia, Vietnam, Afghanistan and Ethiopia. Participants with uncomplicated vivax malaria were treated with schizonticidal treatment for acute peripheral parasitaemia and then one of three treatment arms to prevent recurrent parasitaemia arising from reactivation of the dormant liver stages (relapses). The three arms included: high dose primaquine (total dose 7 mg/kg) administered either over 14 days (0.5 mg/kg/day) or 7 days (1 mg/kg/day), or placebo [18]. At enrollment all patients agreed to take a complete course of treatment and then be followed up once per month for 12 months. The detailed study design has been reported previously [17].

Results of the study were disseminated to academics and healthcare professionals through conference presentations and publication [18], and to policy makers on national and regional level through the Asia Pacific Malaria Elimination Network (APMEN).

### The dissemination strategy

**Fig. 1:**
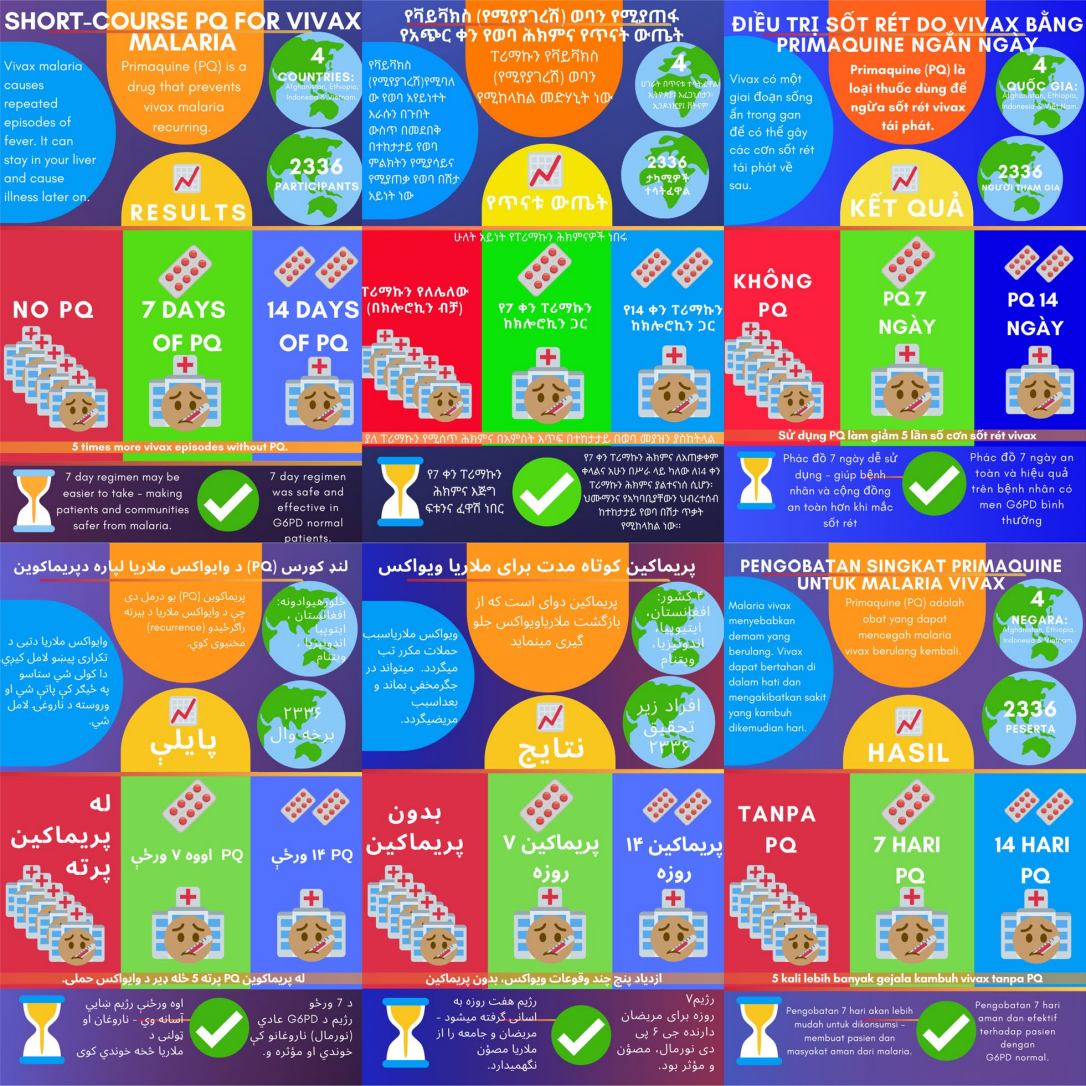
Pictorial summary of study results in English, Amharic, Vietnamese, Dari, Pashto and Bahasa Indonesia

A two phased approach to disseminate study results to participants was developed: (i) simple and easy to understand pictorial information in local languages was provided to all eight study sites and distributed to trial participants (Fig. 1), and (ii) a face-to-face participant group meeting was held at one of the study sites in Ethiopia. Details of the reporting process and lessons learned from the face-to face component are presented.

The site investigator invited a total of 43 participants to participate in the meeting at the research center in Arba Minch, Ethiopia in November 2019. The meeting was attended by: trial participants (n = 33), medical interns (n = 5) and hospital officials (n = 5). Trial participants were selected based on their availability at the time of the meeting as well as their age and gender to ensure good representation of the trial population. In total the participants represented 9% of the total number of participants recruited into the trial at the site. The number of participants was limited by the available space in the meeting location and a limited budget to hold multiple meetings. The entire trial team that supported the clinical trial was present.

At the start of the meeting participants were welcomed and received the pictorial information sheet. The meeting was facilitated by the site investigators, and one of the international investigators. Study participants were acknowledged for their commitment in the study, a summary of the study results was given in lay terms followed by a question and answer (Q & A) session. The meeting was concluded by an informal gathering with food and drink to allow further questions to be asked outside the formal meeting. All participants were reimbursed for any costs incurred by travelling to the venue.

## Lessons learned

During the Q & A session study participants engaged very quickly with the facilitators without further prompting. Main message that emerged are discussed below.

### Participants experience

All study participants who spoke during the meeting expressed their appreciation for the conduct of the trial itself and the process of dissemination of the results. One participant commented that he had felt well informed from the start of his encounter with trial investigators, including the consent process, and during the trial follow up visits. Another participant mentioned that initially she had not wanted to participate in the trial because of the intense follow up, but after consenting it had been a positive experience and she was now happy to hear about the results.

*“I am afraid of professional health staff and of syringes, so first I didn’t want to participate, but then I changed my mind. Everything is worthwhile now.”*

Other participants commented on the positive experience of having less malaria episodes, particularly for their children.

Participant experiences in a clinical trial influence retention [19], which is particularly important in studies with prolonged follow up. Enhancing the trial experience requires consideration of the potential benefits and burdens of research participation for each stage of the trial process. Research burdens associated with clinical trials have psychological, physical and financial implications, that occur both during follow-up and after completion of participation [18]. Failure to anticipate or mitigate the negative impacts of participation has potential to influence future participation in clinical research and willingness to contribute time for longitudinal cohorts [20].

The comments expressed during the meeting may not reflect the entire breadth of experiences participants experienced and a more formal assessment would be necessary to better understand the nuances. It was also unknown whether participants were allocated to the control or intervention arms during the trial and if experiences might have differed between treatment arms. Formal assessments of participants’ experiences are rarely undertaken systematically [21], but such an approach could potentially inform study conduct. Furthermore, it is unclear what methods of dissemination are most appropriate and they are likely to be context and trial specific [21, 22].

### Routine health care versus trial participation

Several participants at the meeting expressed feeling thankful towards the study staff, remarking that the healthcare within the study was superior to that experienced during routine encounters at the hospital. This reinforced the importance of good study conduct for study staff and served as motivation for future studies. However, there is an inherent risk, particularly in resource limited settings, of decreasing satisfaction in already weak public health care systems. One participant expressed this clearly:

*“At the hospital you have to wait 3* *h before someone is treating you, here I got immediate and continuous care”*

Another study participant said:

*“So many children are suffering, the hospital service is bad, the service in research center is much better”*

Although differences in the quality of care during clinical trials compared to routine care have been documented previously, they have not been studied empirically [23, 24]. It is recognized that trials have the potential to benefit facilities, staff and communities through improvement of routine care and physical facilities—ensuring regular supplies and access to essential drugs and clinical equipment. Likewise, they can generate negative effects by diverting resources, such as qualified staff, away from routine settings thus introducing inequities between trial participants and non-participants [25–27]. It remains challenging within clinical trials to retain satisfaction in routine care and at the same time ensure that the impact following the trial completion is positive [25, 28].

### Empowering patients

Several participants expressed the need for more research and their willingness to participate in such research; the incentive being potential improvements in healthcare and thus health outcomes of their communities. One participant said:

*“We need more research also in other diseases like typhoid”*

Another participant raised concerns about the implementation of research findings into practice

*“This research shows we need primaquine and it should be made available”*

These statements reflect an expanding need to empower participants and their communities to be involved in decisions shaping local health and research priorities. The World Health Organization (WHO) defines empowerment as “a process through which people gain greater control over decisions and actions affecting their health” [29]. People acting individually or in groups can play decisive roles to leverage health care policy reform, as evidenced in countries like Australia, the US and Canada who have a long history of patient interest groups influencing policy development [30, 31]. In many African countries however, policy participation by local communities is less common [32], and while influence can be difficult to measure, the general public has limited power or ability to contribute in most public policy decisions [33–35]. Despite this, promotion of patient engagement and advocacy can build trust between communities, healthcare providers and researchers, improving health and reducing disparities. This may for example be done through the establishment of Community Advisory Boards [15]. Ultimately participant empowerment can facilitate the implementation of results into policy development through participatory mechanisms [36].

### Combined session for two audiences

When attempting to align the dissemination of results with the abilities and skills of the meeting attendees, several challenges were faced. The meeting was conducted with trial participants and healthcare staff from the hospital. The content was delivered in a manner directed specifically towards participants with low health literacy, using a visual aid and clear, plain language without medical jargon. The aim was to demystify the results for participants, but also update the hospital staff as a courtesy and to keep them engaged. One challenge for the investigators was finding a suitable level of detail that would not overwhelm the participants with information. While the study staff benefitted from hearing positive experiences about participants in the trial, they also requested a higher level of detail and asked questions often too complex for the participants. Previous research has shown it is particularly important that persons with low health literacy are involved in feedback of clinical care [37], and various models exist for communicating clear results between healthcare providers and study participants [38].

## Limitations

The findings and lessons learned from the dissemination process have several limitations. Firstly, it was not possible to invite all trial participants to attend the meeting. Although a balanced selection was attempted based on age and gender, the cohort that attended, as well as the positive responses received is likely skewed towards participants with higher health literacy, greater interest in the outcomes, and those who had a positive experience during the study. Secondly, it is anticipated that snowballing information cascades occured towards other trial participants and the general community post-dissemination, however it was not possible to confirm this. Furthermore, systematic confirmation about participants’ experiences in the trial were not sought. Findings could be limited by participants emphasizing positive experiences over negative ones due to social desirability bias. Lastly, the level of understanding about the trial results after the meeting was not assessed, so it was not possible to comment on the extent and quality of knowledge transfer.

## Conclusion

This simple, face-to-face dissemination meeting for clinical trial participants in addition to pictorial information was an opportunity for them to gain a better understanding of the trial results and see the value of research participation for the community. Distributing feedback to study participants aids in the transparency and uptake of results, promotes trust, and builds engagement and collaborations with community stakeholders. Further research is needed to design better mechanisms with clear and appropriate communication of research to ensure tangible benefits of dissemination to both researchers and study participants.

## Data Availability

Not applicable.
